# Norepinephrine and Epinephrine Enhanced the Infectivity of Enterovirus 71

**DOI:** 10.1371/journal.pone.0135154

**Published:** 2015-08-07

**Authors:** Yu-Ting Liao, Shih-Min Wang, Jen-Ren Wang, Chun-Keung Yu, Ching-Chuan Liu

**Affiliations:** 1 Center of Infectious Disease and Signaling Research, National Cheng Kung University, Tainan, Taiwan; 2 Department of Microbiology & Immunology, College of Medicine, National Cheng Kung University, Tainan, Taiwan; 3 Department of Emergency Medicine, National Cheng Kung University Hospital, College of Medicine, National Cheng Kung University, Tainan, Taiwan; 4 Department of Medical Laboratory Science and Biotechnology, College of Medicine, National Cheng Kung University, Tainan, Taiwan; 5 Department of Pediatrics, National Cheng Kung University Hospital, College of Medicine, National Cheng Kung University, Tainan, Taiwan; Centro de Biología Molecular Severo Ochoa (CSIC-UAM), SPAIN

## Abstract

**Background:**

Enterovirus 71 (EV71) infections may be associated with neurological complications, including brainstem encephalitis (BE). Severe EV71 BE may be complicated with autonomic nervous system (ANS) dysregulation and/or pulmonary edema (PE). ANS dysregulation is related to the overactivation of the sympathetic nervous system, which results from catecholamine release.

**Objective:**

The aims of this study were to explore the effects of catecholamines on severe EV71 infection and to investigate the changes in the percentages of EV71-infected cells, virus titer, and cytokine production on the involvement of catecholamines.

**Study Design:**

Plasma levels of norepinephrine (NE) and epinephrine (EP) in EV71-infected patients were measured using an enzyme-linked immunoassay. The expression of adrenergic receptors (ADRs) on RD, A549, SK-N-SH, THP-1, Jurkat and human peripheral blood mononuclear cells (hPBMCs) were detected using flow cytometry. The percentages of EV71-infected cells, virus titer, and cytokine production were investigated after treatment with NE and EP.

**Results:**

The plasma levels of NE and EP were significantly higher in EV71-infected patients with ANS dysregulation and PE than in controls. Both α_1A_- and β_2_-ADRs were expressed on A549, RD, SK-N-SH, HL-60, THP-1, Jurkat cells and hPBMCs. NE treatment elevated the percentages of EV71-infected cells to 62.9% and 22.7% in THP-1 and Jurkat cells, respectively. Via treatment with EP, the percentages of EV71-infected cells were increased to 64.6% and 26.9% in THP-1 and Jurkat cells. The percentage of EV71-infected cells increased upon NE or EP treatment while the α- and β-blockers reduced the percentages of EV71-infected cells with NE or EP treatment. At least two-fold increase in virus titer was observed in EV71-infected A549, SK-N-SH and hPBMCs after treatment with NE or EP. IL-6 production was enhanced in EV71-infected hPBMCs at a concentration of 10^2^ pg/mL NE.

**Conclusion:**

The plasma levels of NE and EP elevated in EV71-infected patients with ANS dysregulation and PE. Both NE and EP enhanced the percentages of infected cells and virus titers in EV71 infection *in vitro*. NE and EP may play a role in the pathogenesis of EV71 BE complicated with ANS dysregulation and PE.

## Introduction

Human enterovirus 71 (EV71) is a positive-sense single-stranded RNA virus that belongs to the genus *Enterovirus* (human enterovirus A) of the family *Picornaviridae*. In 1969, EV71 was first isolated from a child with encephalitis in the United States. Outbreaks have since been reported worldwide, including reemerging epidemics in Asia Pacific [1, 2]. Infection with EV71 involves diverse clinical features, including mild febrile illness, characteristic cutaneous diseases such as hand, foot, and mouth disease (HFMD) and herpangina, and severe neurological syndromes. EV71 is a neurotropic virus that can cause acute flaccid paralysis, brainstem encephalitis (BE), autonomic nervous system (ANS) dysregulation, pulmonary edema (PE), and cardiopulmonary failure among children [1–5]. The EV71 BE has been categorized into three stages according to disease severity, namely uncomplicated BE, ANS dysregulation, and PE [3, 4, 6]. ANS dysregulation is defined by the sympathoexcitation caused by the destruction of the brainstem of the central sympathetic network by EV71. A previous study demonstrated that patients with EV71 BE and ANS dysregulation presented tachycardia and hypertension clinically, and elevated plasma levels of norepinephrine (NE) and epinephrine (EP) [7]. Abundant catecholamines and strong central nervous system (CNS) inflammatory responses aggravate cytokine storm and pulmonary vascular permeability, causing PE [1, 8]. The number of CD4^+^ and CD8^+^ T cells and nature killer (NK) cells decreased as the patients advanced from ANS dysregulation to PE [9]. Plasma levels of IFN-γ-induced protein (IP)-10, monocyte chemoattractant protein (MCP)-1, monokine induced by IFN-γ (MIG), IFN-γ, IL-8, IL-10, and IL-13 are significantly elevated in patients with PE [6, 9, 10, 11]. Both systemic inflammatory responses and CNS inflammatory responses are critical in the pathogenesis of EV71 PE. However, the role of catecholamine in the pathogenesis of EV71 infection has not been delineated.

Catecholamines are neurotransmitters and neuromodulators, including NE, EP, and dopamine (DP), that influence neuroendocrine-immune interaction during stress and infection to form a balanced immune response [12]. The main neurotransmitters of sympathetic efferent at the target organs are NE and EP. By targeting α- (α_1_ and α_2_) or β- (β_1_, β_2_ and β_3_) ADRs, locally released circulating NE and EP can innervate lymphoid organs and modulate cytokine and antibody production [12, 13]. The patterns of ADR expression are different on various cells and may upregulate or downregulate cell development or response to stimuli. The β_2_-ADR is the major subtype on immune cell and is considered the main mediator of the immune responses of NE and EP [14, 15, 16, 17]. Moreover, NE and β_2_-ADR stimulation promote IFN-γ secretion, which positively affects CD4^+^ naïve T- and Th1 cell-mediated responses [18, 19, 20, 21]. The α_1_-ADR stimulation positively regulates LPS-initiated IL-1β production and enhances IL-10 production in human mononuclear cells [22]. Catecholamines may induce stimulatory and/or inhibitory effects depending on the concentration of catecholamines and the time of ADR stimulation in relation to the stage of immune responses. Although elaborate network ensures a balance between the nervous and immune systems, NE and EP influence extensively on the progression of infectious and immune-mediated diseases. Several studies have indicated that NE may increase mortality and prolong recovery time because of less monocyte recruitment and higher bacterial loads [15]. Furthermore, NE may accelerate human immunodeficiency virus replication in infected PBMCs by β-ADR and suppress IFN-γ and IL-10 production [13, 14, 15]. Therefore, catecholamines may affect pathogen-induced immune responses. In this study, the catecholamine levels in EV71-infected patients were analyzed. The role of catecholamine in EV71 infectivity and cytokine expression were investigated by using in vitro and ex vivo models.

## Methods

### Clinical specimens

Enrolled EV71-infected patients were confirmed virologically. Though small sample sized, patients were divided into two groups: (1) uncomplicated BE (n = 2) and (2) BE with ANS dysregulation and BE complicated with PE (n = 7). Venous blood samples were collected into EDTA evacuated tubes (BD Vacutainer, USA) for NE and EP assay. Plasma was separated using centrifugation at 1200 *g* for 10 minutes and was stored at -70°C until analyzed. The Clinical Research Ethics Committee of the National Cheng Kung University Hospital approved the study protocol. Written informed consent was obtained from the parents or guardians of participants.

### Cells and virus

Cells of A549 (human lung carcinoma) (BCRC No. 60074; October 2008), RD (human rhabdomyosarcoma) (BCRC No. 60113; October 2008), SK-N-SH (human neuroblastoma) (ATCC No. HTB-11; December 2008), HL-60 (human promyelocytic leukemia) (BCRC No. 60027; March 2009), THP-1 (human monocytic leukemia) (BCRC No. 60430; October 2008), and Jurkat (human T cell leukemia) (BCRC No. 60424; January 2009) were maintained in Dulbecco's Modified Eagle Medium (DMEM) or RPMI 1640 medium (Life Technologies Gibco, USA) with heat-inactivated 10% fetal bovine serum (FBS) (Biological Industries, Israel) according to the instructions of the manufacturer [23, 24, 25]. The hPBMCs were isolated from whole EDTA blood by using ficoll-hypaque gradient centrifugation (GE Healthcare Bio-Sciences, Sweden) and cultured in RPMI 1640 medium (Life Technologies Gibco, USA) containing 10% fetal bovine serum and 2 mM L-glutamine plus 100 IU of penicillin, 100 μg of streptomycin, 250 ng of amphotericin B, and 50 μg of gentamicin (Life Technologies Gibco, USA) per mL. The A549, RD, HL-60, THP-1, Jurkat were purchased from Bioresource Collection and Research Center (Hsinchu, Taiwan). The SK-N-SH was obtained from America Type Culture Collection (Manassas, VA, USA). The Virology Laboratory of National Cheng Kung University Hospital provided an EV71 strain (Taiwan/4643/98). To preserved virus stocks, viruses were propagated for one more passage in RD cells. Virus titer determination was performed using plaque assay, as described in a previous study [26].

### NE and EP measurements

The plasma concentration of NE and EP were measured using a commercial immunoassay kit (Labor Diagnostika Nord GmbH & Co. KG, Germany). A cis-diol-specific affinity gel was used to extract 100 μL of plasma, which was then acylated and derivatized enzymatically. Extracted supernatants were quantified using a competitive enzyme-linked immunoassay according to the instructions of the manufacturer. To ensure the quality of measurements, the control samples were included in the assay. The intra-assay coefficient of variations was 8.4% to 15.6% for NE and 9.3% to 17.1% for EP. The analytical sensitivity in this assay was 2.4 pg/mL for NE and 3.6 pg/mL for EP.

### PMA or DMSO stimulation

Jurkat and THP-1 cells (5 × 10^5^ cells/mL) were treated with 5 ng/mL of phorbol 12-myristate 13-acetate (PMA; Sigma-Aldrich Co. LLC, USA) for 24 and 48 hours, and HL-60 cells were treated with DMSO (13 μL in 1 mL cell suspension) for 7 days. The Jurkat cells were then matured into activated T cells; the THP-1 cells were changed into adherent form cells and matured into macrophage-like cells; and the HL-60 cells were activated into neutrophil-like cells. The activation of cells was confirmed using flow cytometry (BD FASCalibur, USA) to examine the expression of CD3, CD69, CD14, and CD11b (BD Pharmingen, USA) [23, 24, 25].

### EV71 infection and catecholamine treatment *in vitro*

Cells (1 × 10^5^ cells/well) in a 48-well plate were infected by EV71 with multiplicities of infection (MOI) of 1, 5, or 10. After incubation, infected cells were treated with NE (Sanofi Winthrop Industrie SA, France), EP (China Chemical & Pharmaceutical Co., TW), dopamine (Orion Co., Finland), or dobutamine (Synmosa Biopharma Co., TW) at concentrations of 1, 10^1^, 10^2^, 10^3^, 10^4^, 10^5^, and 10^6^ pg/mL, separately. Blocking ADR assays were performed by adding 1 μM phentolamine hydrochloride (α-blocker; Sigma-Aldrich Co. LLC, USA) or 1 μM propranolol hydrochloride (β-blocker; Sigma-Aldrich Co. LLC, USA) 1 hour before adding catecholamines. All catecholamines and blockers were diluted with fresh culture medium containing 2% FBS into conditional concentrations.

### Immunofluorescent staining

EV71-infected and mock control cells on circular-shaped glass slides (12 mm) were fixed in 4% paraformaldehyde/phosphate-buffered saline (PBS) for 10 minutes at room temperature and then washed twice with PBS. Cells were incubated for 30 minutes at room temperature with a 1:300 dilution of anti-EV71 VP1 monoclonal antibody (MAb979; Millipore, Billerica, MA, USA). A 1:200 dilution of alexa 488-conjugated goat anti-mouse IgG antibodies (Life Technologies Gibco, USA) was added and counterstained with DAPI (Vector Laboratories, California, USA). The cells and viral particles were observed using fluorescent microscopy. Staining β_2_-ADR (Abnova Corporation, Taiwan) on THP-1 followed the same procedure.

### Flow cytometry

To detect ADRs, cells were stained with a 1:300 dilution of rabbit anti-α_1A_ ADR or β_2_-ADR antibodies (Abnova Corporation, Taiwan). After incubation, a 1:200 dilution of alexa 488-conjugated goat anti-rabbit IgG antibody (Life Technologies Gibco, USA) was added. PBS stained cells and secondary antibody stained cells were performed as negative controls in each experiment. To measure the percentages of infected cells, cells were permeabilized with 70% ethanol at -20°C and washed with PBS. Each infection experiment measured by staining a 1:300 dilution of anti-EV71 monoclonal antibody (MAb979; Millipore, Billerica, MA, USA) and a 1:200 dilution of alexa 488-conjugated goat anti-mouse IgG antibody (Life Technologies Gibco, USA). Mock-infected cells were used as negative control to gate infected cells. The RD cells were infected with EV71 as positive control in each infection replica. All stained cells were analyzed using flow cytometry (BD FASCalibur) and WinMDI 2.8 software.

### Plaque assay

The RD cells (1.5 x 10^5^ cells/well) were seeded in 24-well plates, incubated at 37°C for 16–18 hrs and then removed culture medium. Each 10-fold serial dilution of the samples was added to wells. After absorption for 1 hr at 37°C, overlay medium containing 2% FBS and 0.8% methylcellulose were added and incubated at 37°C for 72 hrs. The overlay medium was discarded and stained with solution containing 4% formaldehyde and 1% crystal violet in PBS at room temperature for 1 hr. The plates were washed with flowing water and dried to count plaques (unit: PFU/mL).

### Cytokines assay

Cell supernatants were collected and stored at -70°C before assay. Subsequently, IL-6, IL-8, IL-12p70, IL-1β, and IL-10 were detected using cytometric beads array (CBA) kits (BD Pharmingen, USA) according to the instructions of the manufacturer. Five populations of beads with distinct fluorescence intensities were coated with cytokine-specific capturing antibodies. The cytokine-captured beads were then mixed with 30 μL of supernatant and phycoerythrin-conjugated detection antibodies to form sandwich complexes. After incubation, washing, and the acquiring of fluorescence data, the results were generated using BD FCAP array software (version 1.0.8). The limitation of CBA were 2.5 pg/mL for IL-6, 3.6 pg/mL for IL-8, 1.9 pg/mL for IL-12p70, 7.2 pg/mL for IL-1β, and 3.3 pg/mL for IL-10.

### Statistical analysis

Data were compiled in GraphPad Prism 6 (GraphPad Software, Inc.). Continuous data were tested using analysis of variance or the Mann-Whitney U test. All analyses were performed using SPSS software (version 17.0; Chicago, IL). Differences were considered significant at *P* < .05.

## Results

### Plasma level of NE and EP in EV71-infected patients

**Fig 1:**
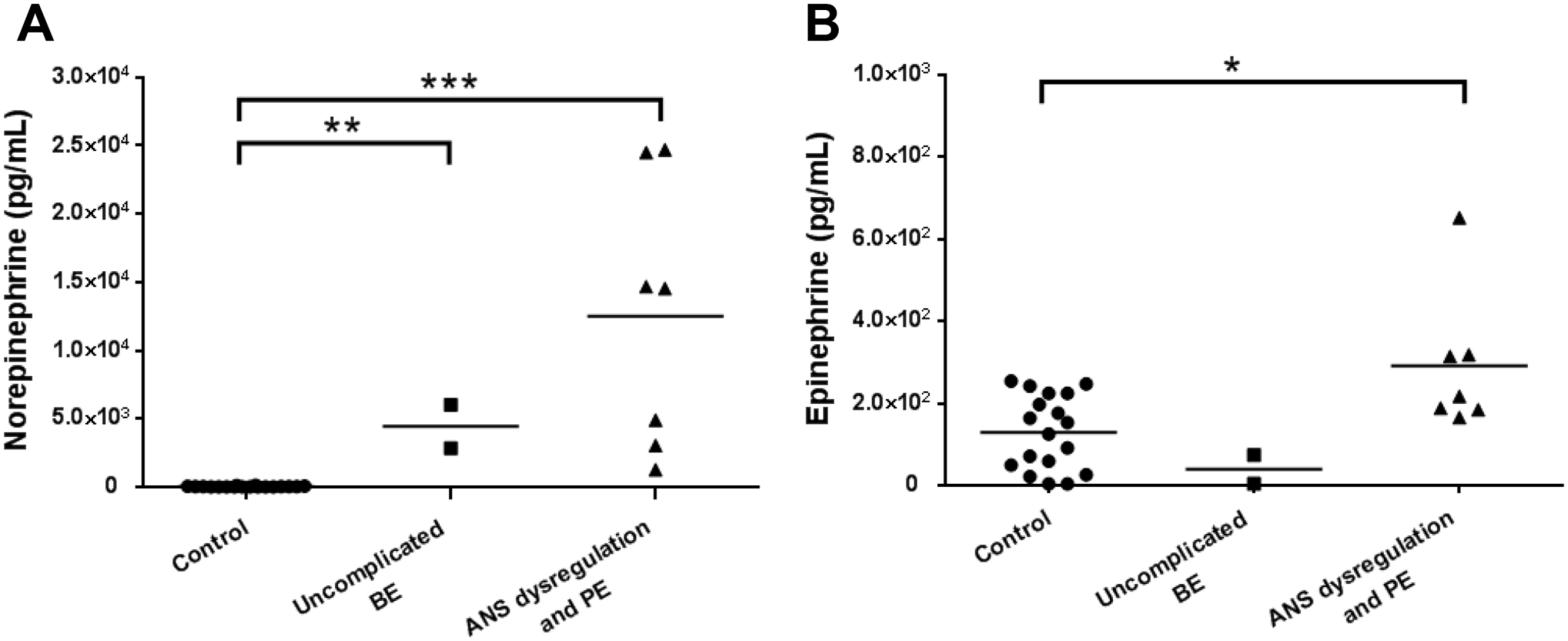
Plasma levels of norepinephrine (NE) and epinephrine (EP) in EV71-infected patients and control subjects. Cases were categorized into three groups: control (n = 18), uncomplicated BE (n = 2) and ANS dysregulation or PE (n = 7). The plasma levels of NE were significantly higher in EV71-infected patients with uncomplicated BE, and ANS dysregulation and PE than in controls (A). Plasma concentrations of EP were significantly higher in EV71-infected patients with ANS dysregulation and PE than in controls (B). BE, brainstem encephalitis; ANS dysregulation, autonomic nervous system dysregulation; PE, pulmonary edema. *, *P* < 0.05; **, *P* < 0.01; and ***, *P* < 0.001.

Plasma levels of NE were significantly higher in patients with uncomplicated BE (4425.0 ± 1590.0 pg/mL, range: 2835–6015 pg/mL, *P* < .01), and ANS dysregulation and PE (12510.0 ± 3699.0 pg/mL; range: 1260–24690 pg/mL, *P* < .001) than in the controls (30.2 ± 8.7 pg/mL; range: 2.4–124.0 pg/mL) (Fig 1A). Moreover, the plasma levels of EP in patients with ANS dysregulation and PE (291.1 ± 64.6 pg/mL; range: 165–652 pg/mL, *P* < .05) were higher than controls (129.5 ± 21.3 pg/mL; range: 3.6–254.0 pg/mL) (Fig 1B). Whereas, there was no significant difference between levels of EP in uncomplicated BE patients (38.8 ± 35.2 pg/mL; range: 3.6–74.0 pg/mL, *P* = .0556) and ANS dysregulation and PE patients.

### Expression of α_1A_- and β_2_-ADRs

**Fig 2:**
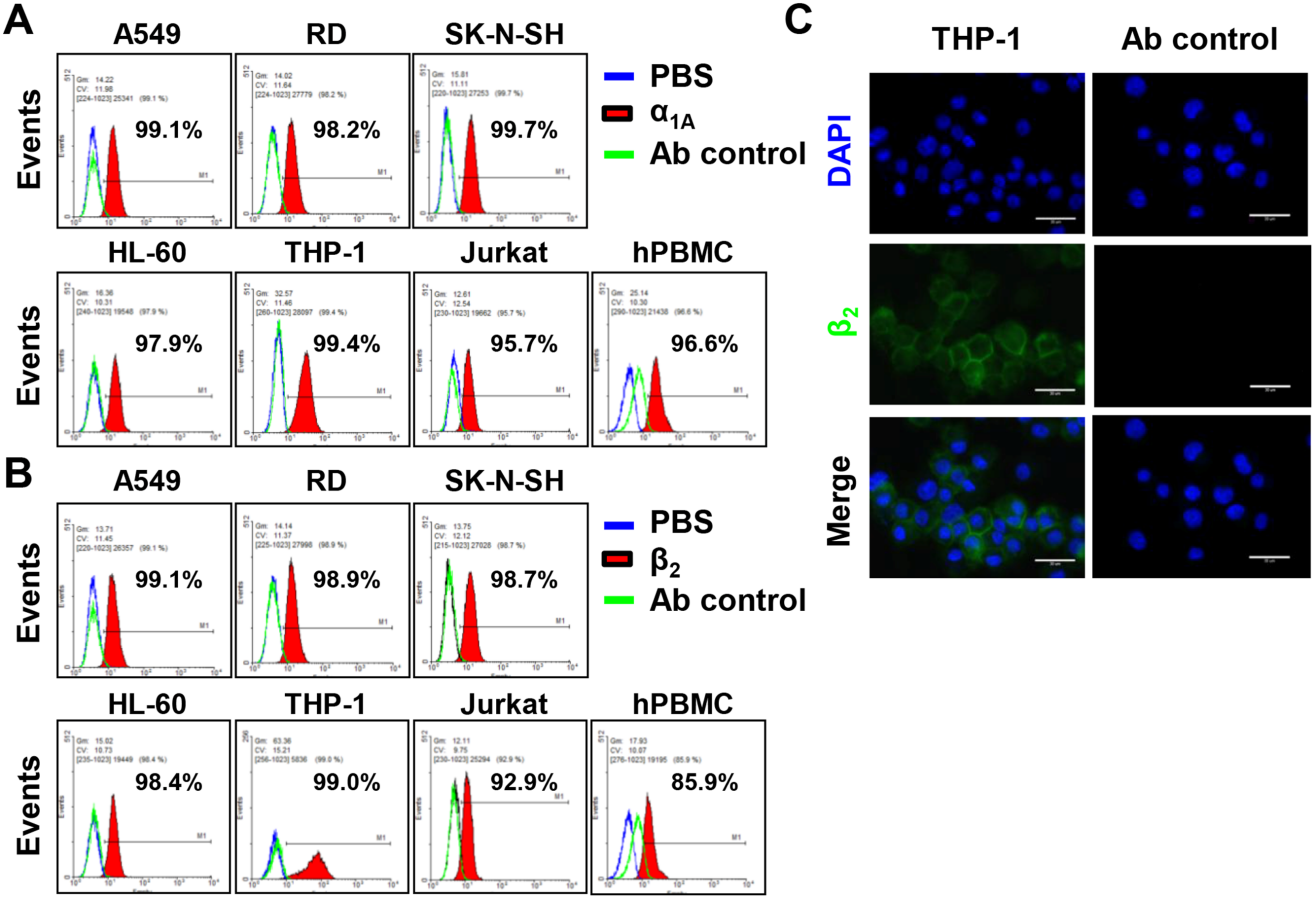
Expression of α_1A_- and β_2_-adrenergic receptor on cell lines. Cells were stained with (A) anti-α_1A_ ADR or (B) β_2_-ADR antibodies. After incubation, washing and centrifugation, alexa 488-conjugated gaot anit-rabbit IgG antibody was added and determined by flow cytometry (red filled region). PBS stained cells (blue lines) and secondary antibody stained cells (green lines) were showed as negative controls in each experiment. A549, RD, SK-N-SH, HL-60, THP-1, Jurkat and hPBMCs exhibited (A) α_1A_- and (B) β_2_-ADRs. (C) The expression of β_2_-ADR on PMA-induced THP-1 cells were observed by immunofluorescent microscopy. The green fluorescence represented β_2_-adrenergic receptor. DAPI (blue) indicated cell nucleus. hPBMC, human peripheral blood mononuclear cells. The data were represented from three independent experiments.

Two major types of ADRs, namely α_1A_- and β_2_, were detected on various cell lines. Nonimmune cells included RD, A549, and SK-N-SH that exhibited α_1A_- and β_2_-ADRs (Fig 2A and 2B). The HL-60, THP-1, and Jurkat cells were induced into neutrophil-like cells, macrophage, and activated T-cells by PMA or DMSO stimulation, respectively. After induction, the HL-60, THP-1, Jurkat cells expressed α_1A_- and β_2_-ADRs (Fig 2A and 2B). Moreover, hPBMCs also possessed α_1A_-ADR (96.6%) and β_2_-ADR (85.6%) (Fig 2A and 2B). The expression of β_2_-ADR was observed on PMA-induced THP-1 cells by using an immunofluorescent microscope (Fig 2C).

### Effects of NE and EP on the percentage of infected cells

**Fig 3:**
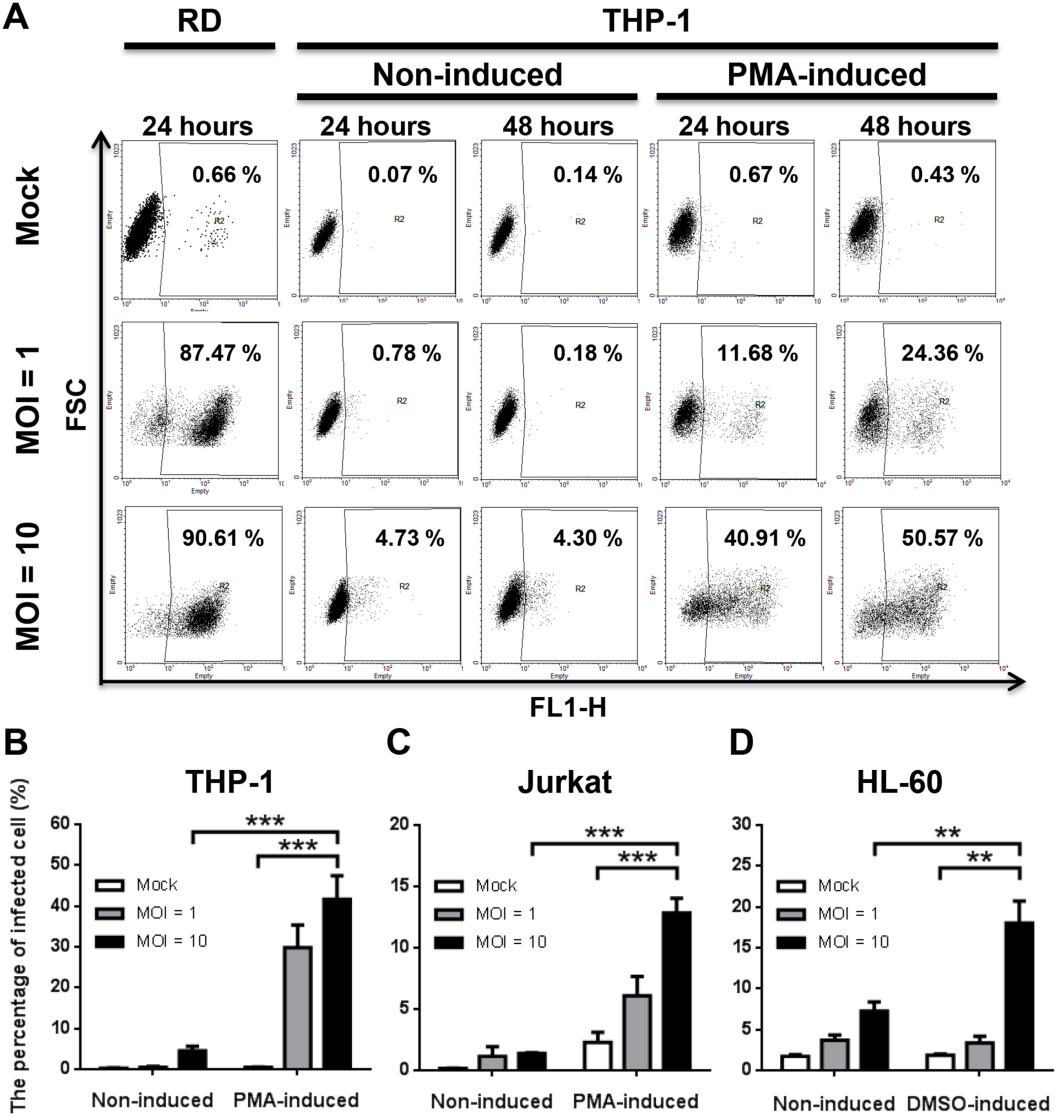
Effects of PMA or DMSO stimulation on immune cell of EV71 infection. (A, B) PMA-induced or non-induced THP-1 cells were infected with EV71 (MOI = 1 and 10). RD cells were infected with EV71 (MOI = 1 and 10) for 24 hrs as positive control. (C) PMA-induced Jurkat and (D) DMOS-induced HL-60 cells were treated and analyzed. The percentages of EV71-infected cells were higher in induced cells than in non-induced cells. The data are shown as mean ± SEM of three independent experiments. ***, *P* < 0.001 analyzed by one-way ANOVA and Post-Hoc Tukey test.

**Fig 4:**
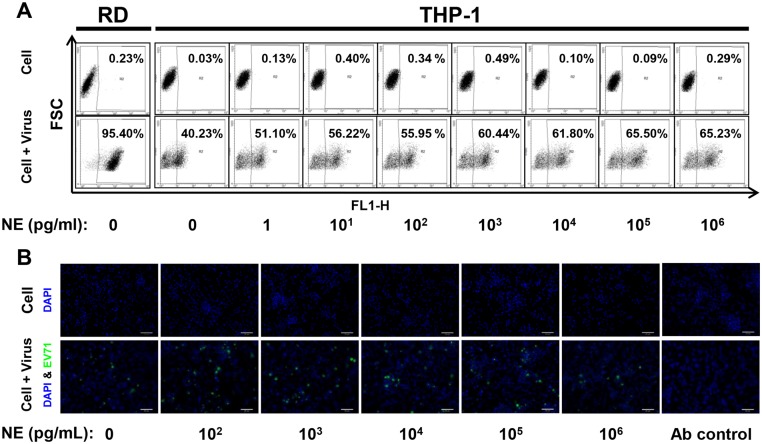
EV71 infectivity after NE treatment on induced THP-1. (A) PMA-induced THP-1 were infected with EV71 (MOI = 10) for 24 hrs and then treated with NE for 24 hrs. The RD cells were infected with EV71 (MOI = 10) for 24 hrs as positive control in each infection replica. Mock-infected cells were used as negative control to gate infected cells. The percentages of EV71-infected THP-1 were increased after NE treatment. (B) Viral particles (green fluorescence) were observed on NE treated and EV71-infected THP-1 using immunofluorescent microscopy. Mock-infected and non-NE-treated THP-1 and secondary antibody stained THP-1 were performed as negative controls. DAPI (blue) staining indicated cell nucleus. The data were represented from three independent experiments.

**Fig 5:**
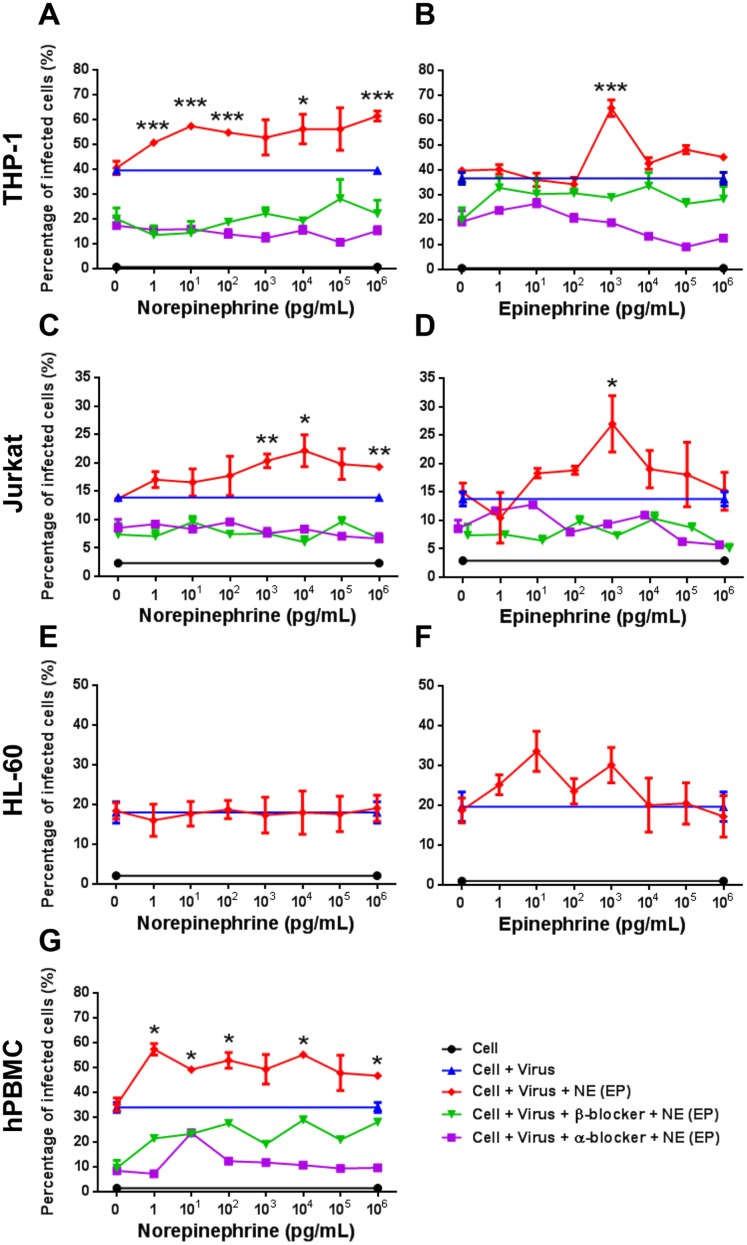
Effects of NE and EP on EV71 infectivity of THP-1, Jurkat, HL-60 and hPBMCs. Induced (A, B) THP-1, (C, D) Jurkat and (E, F) HL-60 were infected with EV71 (MOI = 10) for 24hrs and then treated with NE and EP for 24hrs. The α-blocker (1 μM) and β-blocker (1 μM) treatment were performed 1 hr before adding NE or EP. The percentages of EV71-infected cells were determined by using flow cytomerty. (G) The same infection and NE treatment conditions were performed on hPBMCs. NE or EP treatment enhanced the percentages of EV71-infected cells in THP-1, Jurkat and hPBMCs, but not in HL-60. Both α-blocker and β-blocker reduced the percentages of EV71-infected cells with NE and EP treatment. The data are shown as mean ± SEM of two (G) or three (A-F) independent experiments. *, *P* < 0.05; **, *P* < 0.01; and ***, *P* < 0.001 compared to virus only group and analyzed by one-way ANOVA and Post-Hoc Tukey test. α-blocker, phentolamine hydrochloride; β-blocker, propranolol hydrochloride.

To demonstrate the effects of catecholamines in EV71 infection in vitro, NE and EP were used to treat EV71-infected cells. First, the EV71 infectivity on non-induced and induced cells was tested in different MOI (Fig 3A). After 48 hours post-infection, the percentages of EV71-infected THP-1, Jurkat and HL-60 cells at MOI of 10 showed significant elevation after PMA or DMSO induction compared to non-induced and mock cells (Fig 3B, 3C and 3D; *P* < .001). This indicated that THP-1, Jurkat and HL-60 under induced differentiation were more susceptible to EV71 infection. Thus, induced THP-1 cells were infected with EV71 (MOI = 10) and treated with NE. Cells were permeablized before anti-EV71 VP1 monoclonal antibody staining and the percentage of infected cells were assessed using flow cytometry. After NE treatment, the percentages of EV71-infected THP-1 cells increased from 40.23% to 51.10–65.50% in comparison with non-NE-treated infection replicas (Fig 4A). RD cells were infected with EV71 (MOI = 10) as positive control which showed 95.40% of infected cells (Fig 4A). Viral particles (green fluorescence) were observed on EV71-infected THP-1 with or without NE treatment using immunofluorescent microscopy (Fig 4B). Therefore, induced Jurkat and HL-60 cells were infected by EV71 (MOI = 10) and performed consistent experimental conditions of NE and EP treatment. The percentages of EV71-infected THP-1 cells were significantly enhanced at the concentrations of 1, 10^1^, 10^2^, 10^4^ and 10^6^ pg/mL NE treatment, in comparison with non-NE-treated cells (Fig 5A; *P* < .05, respectively). The percentages of infected THP-1 cells with NE treatment were increased from 39.4 ± 1.5% (EV71 only) to 62.9 ± 2.3% (Fig 5A). The percentages of EV71-infected Jurkat cells significantly increased to 22.7 ± 2.8% from 13.9 ± 0.5% (EV71 only) at the concentrations of 10^3^, 10^4^ and 10^6^ pg/mL of NE treatment (Fig 5C; *P* < .05, respectively). At the concentrations of 10^3^ pg/mL EP treatment, the percentages of EV71-infected cells in THP-1 (64.6 ± 3.4% vs. 36.8 ± 1.8%; Fig 5B; *P* < .001) and Jurkat (26.9 ± 4.9% vs. 13.9 ± 1.0%; Fig 5D; *P* < .05) cells significantly increased in comparison with EV71-infected cells without EP treatment. However, NE or EP treatment did not significantly change the percentages of EV71-infected HL-60 cell (Fig 5E and 5F). To further confirm these findings, two types of ADR blockers, nonselective α-blocker (phentolamine hydrochloride) and β-blocker (propranolol hydrochloride), were used before NE and EP treatment. Both α-blocker and β-blocker treatment decreased the percentages of EV71-infected cells (Fig 5; *P* < .05). Furthermore, NE treatment with conditional concentrations (1, 10^1^, 10^2^, 10^4^ and 10^6^ pg/mL) showed that the percentages of EV71-infected hPBMCs were enhanced from 36.2 ± 2.0% (EV71 only) to 57.2 ± 2.3% (*P* < .05), which were also reduced by α- or β-blockers (Fig 5G; *P* < .05). These findings demonstrate that catecholamines play roles in the infectivity of EV71. The ADRs were involved in the interaction between catecholamine and EV71 infection.

### Virus titer changes after NE or EP treatment

**Fig 6:**
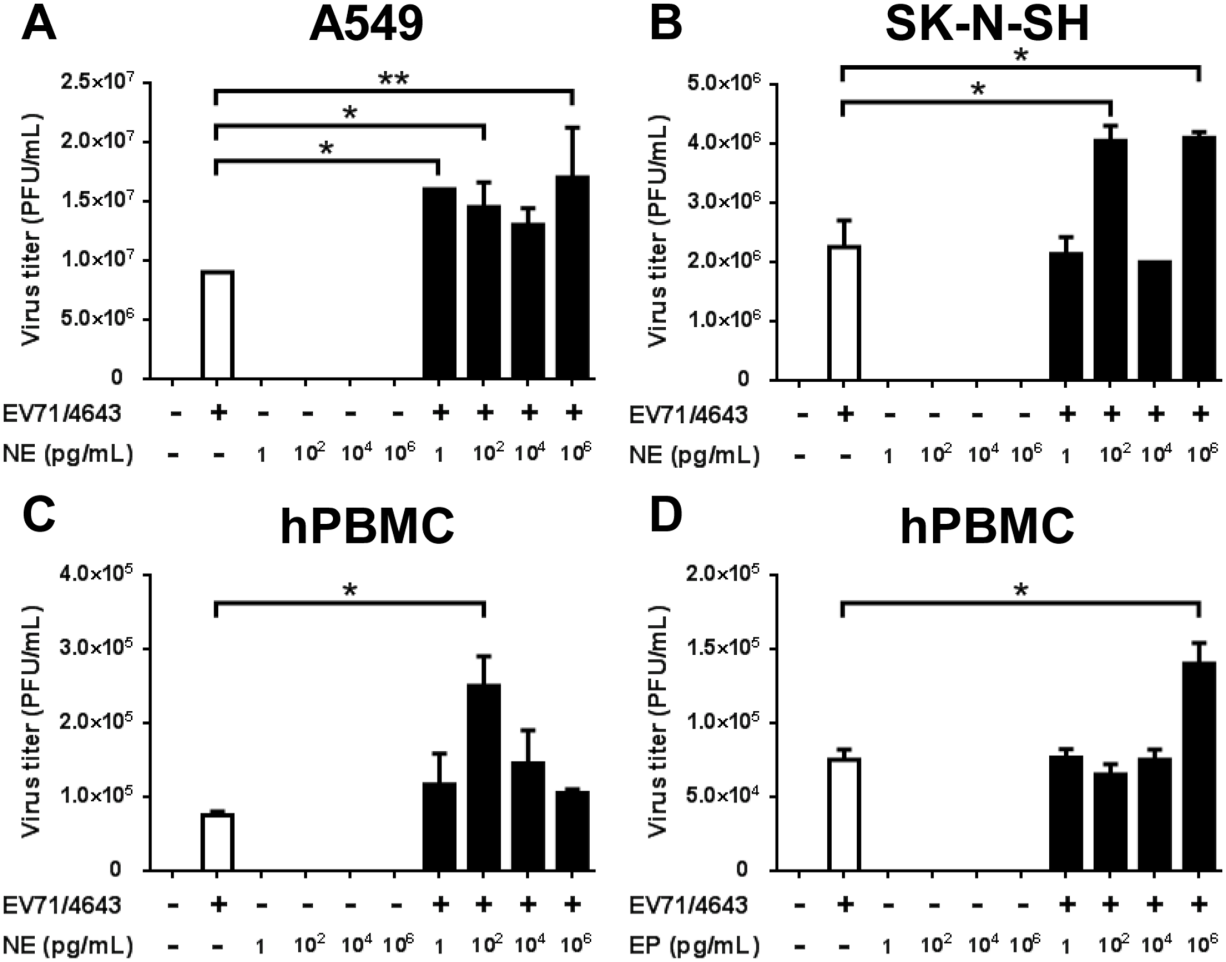
Virus titer changes from NE or EP treatment on A549 cells, SK-N-SH and hPBMCs. (A) A549 cells were infected with EV71 (MOI = 5) for 24 hrs and then treated with NE for 12 hrs. (B) SK-N-SH cells were infected with EV71 (MOI = 1) for 18 hrs and then treated with NE for 6 hours. (C, D) hPBMCs were infected with EV71 (MOI = 10) for 24 hrs and then treated with NE and EP for 24 hrs. The virus titers were significantly higher in (A) EV71-infected A549 cells and (B) SK-N-SH with various concentrations of NE than in EV71-infected cells without NE treatment. Both (C) NE and (D) EP elevated virus titers in EV71-infected hPBMCs at concentrations of 10^2^ and 10^6^ pg/mL, respectively. The data are shown as mean ± SEM of three independent experiments. White bar: virus only; black bar: virus plus NE or EP. *, *P* < 0.05; and **, *P* < 0.01 compared to virus only group and analyzed by one-way ANOVA and Post-Hoc Tukey test.

NE and EP increased the percentages of EV71-infected cells in the in vitro and ex vivo model. To investigate whether catecholamines could affect viral replication, virus titers were assessed by plaque assay. Virus titers were 2-fold higher in EV71-infected A549 cells at the concentrations of 1, 10^2^, and 10^6^ pg/mL NE treatment than in EV71-infected cells without NE treatment (Fig 6A; *P* < .05). Virus titers showed 2-fold increase at the concentrations of 10^2^ and 10^6^ pg/mL NE treatment in SK-N-SH (Fig 6B; *P* < .05). In EV71-infected hPBMCs, both NE and EP led to 3- and 2- fold elevations of virus titers at concentrations of 10^2^ and 10^6^ pg/mL compared to EV71 only, respectively (Fig 6C and 6D; *P* < .05). These findings indicated that NE and EP increase EV71 virus titer in selected cells expressing ADRs.

### Cytokine expression with NE treatment

**Fig 7:**
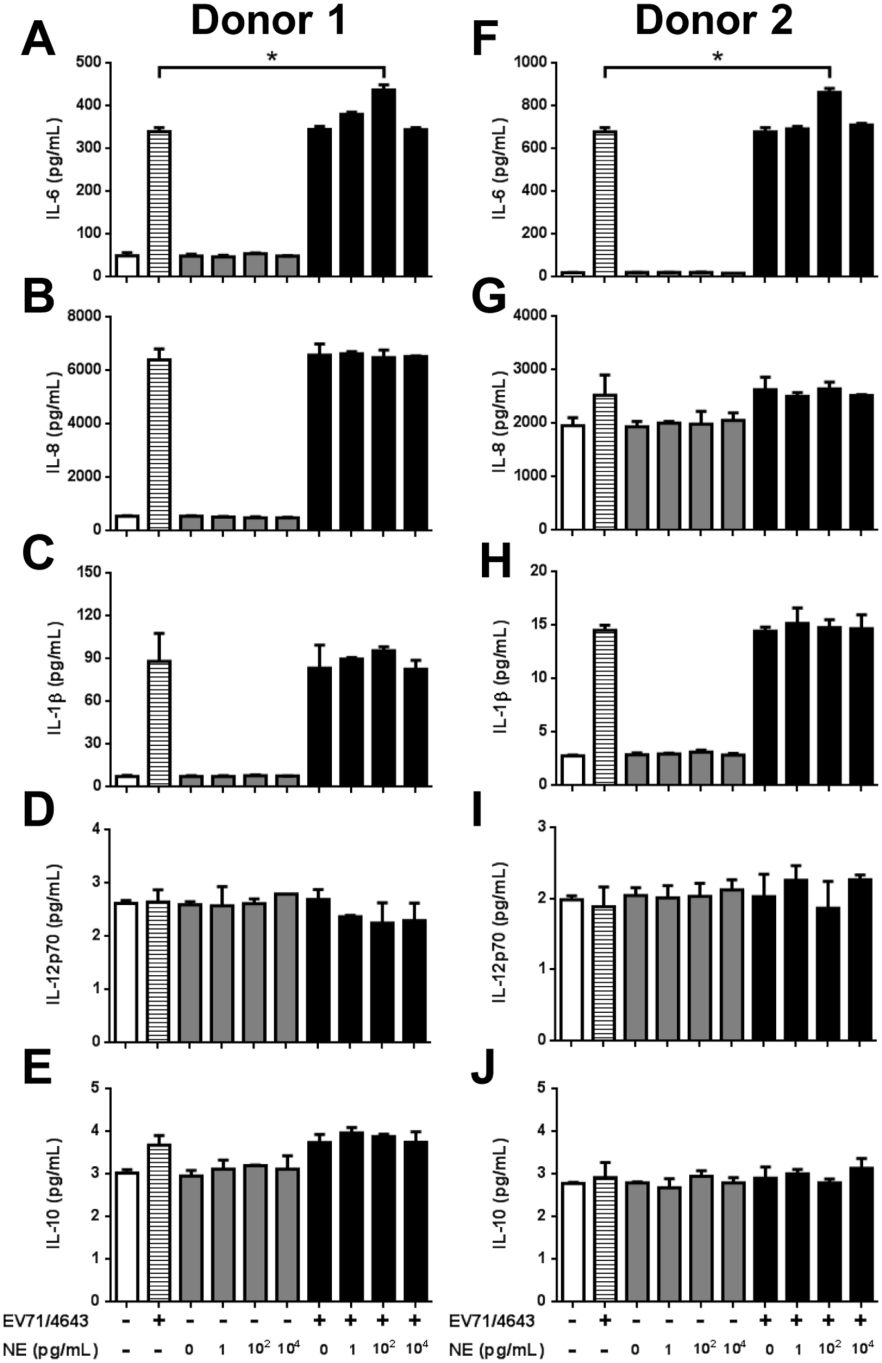
Cytokine expression of hPBMCs after NE treatment. The hPBMCs were infected with EV71 (MOI = 10) for 24hrs and then treated with NE for 24 hrs. The supernatants were collected and measured (A, F) IL-6, (B, G) IL-8, (C, H) IL-1β, (D, I) IL-12p70 and (E, J) IL-10 by cytometric beads array. (A, F) The levels of IL-6 were significantly increased at the concentration of 10^2^ pg/mL NE treatment in EV71-infected hPBMCs compared to infected cells without NE treatment. (B, G) IL-8, (C, H) IL-1β, (D, I) IL-12p70, and (F, J) IL-10 did not significantly change on NE-treated EV71-infected hPBMCs. The data are shown as the mean ± SEM of two independent experiments of each donor. White bar: mock; striated bar: virus only; gray bar: NE only; black bar: virus plus NE. *, *P* < 0.05 compared to virus only group and analyzed by one-way ANOVA and Post-Hoc Tukey test.

Since cytokines are involved in EV71 pathogenesis, IL-6, IL-8, IL-1β, IL-12p70, and IL-10 were measured after treating NE in EV71-infected hPBMCs, which were isolated from two healthy children (Fig 7). The results revealed that IL-8, IL-12p70, IL-1β, and IL-10 did not significantly change in NE-treated and EV71-infected hPBMCs (Fig 7B–7E and Fig 7G–7J). By contrast, the level of IL-6 was significantly increased at the NE concentration of 10^2^ pg/mL in EV71-infected hPBMCs compared to EV71-infected cells without NE treatment (Fig 7A and 7F; *P* < .05).

## Discussion

BE complicated with ANS dysregulation and PE were the neurologic complications in EV71-infected patients. The predominant pathological changes were in the posterior medulla and upper cervical spinal cord [4]. The destruction of the brain stem and tissue causes the overactivation of sympathetic activity and releases catecholamines. Previous studies hypothesized that NE and EP surge into the systemic compartment after brain stem destruction by EV71 [7, 27]. The balanced interactions between sympathetic and parasympathetic nervous systems were not maintained homeostatically in severe EV71 complications. Plasma levels of NE and EP were higher in severe EV71-infected patients than normal subjects [7]. In this study, the plasma levels of NE in EV71-infected patients with uncomplicated BE and with ANS dysregulation and PE were significantly higher than in controls. The plasma levels of EP were significantly higher in EV71-infected patients with ANS dysregulation and PE than in controls. The associations among NE, EP, the percentages of infected cells and virus titers were demonstrated. Our findings suggested that conditional levels of NE and EP enhanced EV71 infectivity and replication. Furthermore, the increased percentages of infected cells and virus titers of EV71 infection by NE and EP via ADRs were exhibited in the ADR blocking assay. Pure inotropic agent, EP, is associated with worse outcomes clinically. It evidenced that therapy with milrinone and dobutamine is effectively to stabilize blood pressure in severe EV71-infected patients [27]. Consequently, the excessive production of catecholamines may involve in the disease deterioration and associate with EV71 infectivity.

Previous researches reported that the presence of subneutralizing concentrations of anti-EV71 antibodies enhances EV71 infection in the monocytic cell line THP-1. Fcγ receptors were proved to mediate partly antibody-dependent enhancement (ADE) in EV71 infection [28]. Furthermore, subneutralizing ADE antibodies increased disease severity, mortality, tissue damage, and cytokine production in an ICR murine model [29]. In the current study, NE and EP enhanced virus titers and the infectivity of EV71 infection in vitro. ADRs may have roles in NE and EP to increase EV71 infectivity. In the pathogenesis of EV71 infection, subneutralizing antibodies, NE, and EP increased infectivity through different receptors and pathways. The interplay of subneutralizing EV71 antibodies with NE and EP in the pathogenesis of EV71 infection warrants further studies.

Nance et al reported that immune cells, including monocyte/macrophage and T and B lymphocytes, expressed β_2_-ADR [12, 30]. Immune (HL-60, THP-1, Jurkat and hPBMCs), muscle (RD), neuron (SK-N-SH), and lung epithelial (A549) cells express α_1A_- and β_2_-ADR. Catecholamines pleiotropically affect the activity of immune cells, primarily through β_2_-ADR [31, 32]. A predominantly deleterious proinflammatory role of NE in sepsis mediated by α_2A_-ADR was also established [33]. The activation of ADRs can alter the production of tumor necrosis factor-α (TNF-α), IL-6, IL-10, IL-12, and chemokine macrophage inflammatory protein 1α (MIP-1α). Previous studies reported that stimulating α_1_-ADRs failed to affect TNF-α and IL-6 production but enhanced IL-10 through EP [34, 35]. However, β-ADRs stimulation elevated plasma levels of IL-6 and IL-10 but suppressed levels of TNF-α, IL-12, and MIP-1α [35, 36, 37]. Lin et al demonstrated that EV71-infected patients complicated with PE had higher blood levels of IL-6. The level of serum IL-6 was the best predictor for EV71 BE with PE [38]. High levels of IL-6 production led to severe tissue damage and death through a neonate mouse model. Anti-IL-6 neutralizing antibodies could reduce mortality, clinical scores, tissue damage and increase immune cell activation [39]. We discovered that NE treatment (10^2^ pg/mL) significantly increased the level of IL-6 in EV71-infected hPBMCs but not levels of IL-8, IL-1β, IL12p70, or IL-10. Therefore, the effects of EP and NE on cytokine expressions and signal transduction pathways deserved further studies.

In this study, the α- and β-blockers were performed to block the effect of NE and EP in EV71 infection. The ADR blocking could reduce the percentages of infected cells that indicated the possibilities of antagonist on severe EV71 infection and implied the importance of ADRs in severe EV71 infection. Moreover, the α- and β-blockers may also influence on mitogen-activated protein kinase (MAPK), NFκB and STAT signaling in the host [40, 41]. To further elucidate the mechanism of catecholamines effects, the blockade of catecholamines in EV71-infected animal model will be investigated.

This study demonstrated that conditional concentrations of NE and EP increase EV71 infectivity and replication. Chemical sympathectomy is a classical model for investigating the influence of sympathetic nervous system activity in host immune responses by 6-hydroxydopamine (6-OHDA) [42, 43]. Grebe et al found that 6-OHDA treatment increased survival rates and reduced lung inflammatory cytokine production and pathology in influenza A virus infection [44]. Furthermore, 6-OHDA augmented CD8^+^ T cell response to both direct and cross-reactive antigens, indicating sympathetic nervous system involvement in altering antiviral adaptive immunity [45]. Moreover, Leo et al demonstrated that peripheral sympathectomy changed cytotoxic T lymphocyte and memory cellular immune responses to herpes simplex virus infections [45, 46]. Our recent study indicated that the depletion of Tregs causes the onset of severe EV71 complications, including ANS dysregulation and PE [47]. Furthermore, NE reduced the frequency of Foxp3^+^ cells and Foxp3 mRNA expression through β_2_-ADR-mediated mechanisms in a concentration and time-dependent manner [48]. Our findings supported the notions that NE and EP are involved in EV71 immunopathogenesis and enhance the infectivity of EV71 through ADRs. NE and EP may be associated with clinical disease severity in complicated EV71 infections.

In summary, the current findings demonstrate that NE and EP enhance EV71 infectivity and inflammation in EV71-associated ANS dysregulation and PE, and affect disease severity. This study highlights the need to further develop sympathoinhibitor as an antiviral and antiinflammatory therapy and for more dose-response and time-course studies in animal models for EV71 infection.

## Supporting Information

S1 Table Characteristics and the expression of NE and EP in EV71-infected patients and control subjects. Eighteen control cases and nine EV71-infected cases were collected. EV71 cases were divided into two groups: uncomplicated BE (n = 2) and ANS dysregulation or PE (n = 7) which were defined by clinical manifestations. The information about sex, age, NE and EP levels were showed in this table.
(DOCX)
