# Supplementary material for: Norepinephrine and Epinephrine Enhanced the Infectivity of Enterovirus 71
Source: PLoS One. 2015 Aug 7;10(8):e0135154. doi: 10.1371/journal.pone.0135154 (PMC4529162; doi:10.1371/journal.pone.0135154)
Supplement: S1 Table — Eighteen control cases and nine EV71-infected cases were collected. EV71 cases were divided into two groups: uncomplicated BE (n = 2) and ANS dysregulation or PE (n = 7) which were defined by clinical manifestations. The information about sex, age, NE and EP levels were showed in this table. (DOCX) [file pone.0135154.s001.docx]

|  | **Control**  **(n = 18)** | | | | **EV71-infected**  **(n = 9)** | | | | |
| --- | --- | --- | --- | --- | --- | --- | --- | --- | --- |
| **No.** | **Sex** | **Age**  **(years)** | **NE**  **(pg/mL)** | **EP**  **(pg/mL)** | **Stage** | **Sex** | **Age**  **(years)** | **NE**  **(pg/mL)** | **EP**  **(pg/mL)** |
| 1 | F | 1.0 | 2.4 | 21.0 | Uncomplicated BE | M | 0.1 | 2835.0 | 74.0 |
| 2 | F | 1.8 | 33.0 | 49.0 | Uncomplicated BE | M | 1.6 | 6015.0 | 3.6 |
| 3 | F | 2.0 | 2.4 | 247.0 | ANS dysregulation and PE | M | 0.2 | 3045.0 | 314.0 |
| 4 | M | 2.1 | 38.0 | 176.0 | ANS dysregulation and PE | F | 0.4 | 14535.0 | 165.0 |
| 5 | F | 2.2 | 109.0 | 71.0 | ANS dysregulation and PE | M | 0.8 | 24690.0 | 318.0 |
| 6 | M | 2.2 | 2.4 | 13.0 | ANS dysregulation and PE | F | 1.3 | 14670.0 | 652.0 |
| 7 | F | 2.3 | 35.0 | 59.0 | ANS dysregulation and PE | F | 2.7 | 24480.0 | 188.0 |
| 8 | F | 2.3 | 2.4 | 224.0 | ANS dysregulation and PE | M | 3.9 | 4890.0 | 217.0 |
| 9 | F | 2.4 | 2.4 | 3.6 | ANS dysregulation and PE | F | 5.5 | 1260.0 | 184.0 |
| 10 | F | 2.4 | 9.0 | 125.0 |  |  |  |  |  |
| 11 | F | 2.5 | 124.0 | 164.0 |  |  |  |  |  |
| 12 | M | 2.7 | 2.4 | 197.0 |  |  |  |  |  |
| 13 | M | 2.8 | 2.4 | 242.0 |  |  |  |  |  |
| 14 | M | 2.9 | 13.0 | 26.0 |  |  |  |  |  |
| 15 | F | 3.0 | 30.0 | 224.0 |  |  |  |  |  |
| 16 | M | 3.2 | 53.0 | 3.6 |  |  |  |  |  |
| 17 | M | 3.3 | 63.0 | 91.0 |  |  |  |  |  |
| 18 | M | 4.2 | 20.0 | 254.0 |  |  |  |  |  |

**S1 Table Characteristics and the expression of NE and EP in EV71-infected patients and control subjects.**
